# Incidence of Heterotopic Ossification in Direct Anterior Approach to Total Hip Arthroplasty with use of Aspirin as Thromboembolic Prophylaxis

**DOI:** 10.51894/001c.12263

**Published:** 2020-06-08

**Authors:** Paul Knapp, Ross Doehrmann, Sanar Yokhana, Syed Rizvi, Judith Boura, David Knesek

**Affiliations:** 1 Orthopaedics Ascension St John Hospital and Medical Center; 2 Orthopaedics Detroit Medical Center; 3 Statistics Ascension Providence

**Keywords:** patient outcomes, post op complications, total joint arthroplasty, anterior approach total hip arthroplasty, total hip arthroplasty, heterotopic ossification

## Abstract

**CONTEXT:**

Heterotopic ossification (H.O.) is a common occurrence after total hip arthroplasty (THA) with significant potential clinical ramifications. Controversy still exists regarding the exact etiology of the disorder, including possible risk factors. Surgical technique, surgical approach, postoperative medication protocols and even thromboembolic prophylaxis have been implicated in the formation of H.O. Our study looked at one institution with a single surgeon performing direct anterior THA (DAA THA) in patients who received aspirin (ASA) as monotherapy for thromboembolic prophylaxis.

**METHODS:**

Patients at a single institution who underwent DAA THA between 2015 and 2019 were identified by CPT code. 45 patients ultimately met inclusion criteria. Postoperative radiographs were analyzed retrospectively for H.O. according to the Brooker classification. Several patient characteristics and comorbidities were statistically analyzed using Chi-square tests, Fisher Exact tests, Wilcox rank sum tests, and Pearson correlation.

**RESULTS:**

12 patients (26.7%) were found to have heterotopic ossification (67% Class 1, 8% Class 2, 25% Class 3, and 0% Class 4); with a median follow up of 35 weeks (range: 12-96). 25% of these patients received ASA 325mg BID while 75% received ASA 81 BID. No statistical differences in development of H.O. were detected among age, gender, BMI, sex, race, diabetes, or NSAID use in the post-operative interval. There were significantly more smokers in the H.O. group (50% vs. 9%, p<0.006).

**CONCLUSIONS:**

Our analysis aimed to quantify the incidence of H.O. with consistency in surgical approach and post-operative protocol. There have been few studies on this topic, and we believe it is very relevant with the increasing use of aspirin in the post-operative protocol for thromboembolic prophylaxis. Our retrospective analysis identified H.O. at rates similar to previous studies in DAA.

## INTRODUCTION

In total hip arthroplasty (THA), the articulation of the proximal femur and acetabulum is resurfaced in order to provide pain relief and restore more normal mechanics/function of the hip joint.^1^ Following THA, a relatively common complication is the development of heterotopic ossification (HO). HO is the process of the soft tissues becoming ossified.^2^ The ossification represents primitive mesenchymal cells in the soft tissues being transformed into osteoblastic tissue. Further development of the tissue leads to the formation of mature lamellar bone.^3^ HO occurs frequently in the femoral neck region, specifically adjacent to the greater trochanter, the area of the proximal femur that the abductor musculature attaches. Monitoring this pathology during clinic-based follow-up is paramount as it tends to progress over time.^4^

Some reported risk factors of HO in THA have included male gender, increased body mass index (BMI), history of HO, hypertrophic osteoarthritis, ankylosing spondylitis, diffuse idiopathic skeletal hyperostosis, Paget's disease, post-traumatic arthritis, rheumatoid arthritis, and decreased preoperative range of motion, and proliferative osteoarthritis.^2,4–6^ Other surgeon-controlled factors such as surgical approach, soft tissue trauma, and prolonged ischemia times have also been considered.^7^

Radiographic evidence of HO has been reported in up to 25 - 40% of THA’s with as many as 10% of patients reporting symptoms affecting functional outcomes and range of motion.^8^ It is important for clinicians to monitor for clinical progression of these lesions as they may have detrimental effects on patient satisfaction and functional outcomes.^9^ Early symptoms of HO may include increasing pain, swelling and stiffness in the effected joint.^10^ Progression is commonly related to a decrease in range of motion on examination with continued pain complaints.

Laboratory studies may be used alongside radiographic and clinical evaluation to diagnose HO. Typically, these labs include a normal serum calcium and phosphorus level, which can help differentiate these lesions from other skeletal and/or endocrine disease processes. Alkaline phosphatase may also be elevated in the initial stages due to increased osteoblast (i.e., bone formation) activity, but typically returns to normal after the bone matures.^10^

Chemoprophylaxis against the formation of HO following THA is common practice among high risk patients.^11^ Prescribed medications include indomethacin, a nonselective cyclooxygenase COX-1 and COX-2 inhibitor, which is routinely administered at an oral dose of 75 mg. twice per day (BID) or 25 mg. three times per day (TID) for three to six weeks postoperatively.^12^ Other Nonsteroidal Anti-Inflammatory drugs (NSAID) and COX-2 inhibitors have also been studied for their effect on HO formation with satisfactory results.^13^ Radiation therapy is another mode of prophylaxis against HO formation with multiple studies claiming no significant difference compared to oral NSAID.^14^

Early stages of HO rarely require any surgical intervention. However, in select patients with substantial functional impairment operative excision may be necessary and helpful. While timing of surgical excision is still debated, there is evidence that patients with significant deficits who have not improved with conservative measures should be offered operative intervention.^15^

Surgeons routinely use Novel Oral Anti-Coagulants (NOAC) or NSAID as post-surgical thromboembolic prophylaxis for prevention of pulmonary embolism (PE) or deep venous thrombosis (DVT) in the post-operative period. These are serious complications of surgery leading to significant morbidity to patients. As more and more physicians transition to the use of Aspirin (ASA) alone for prophylaxis, we may appreciate a decrease in rates of HO.

### Purpose of Study

Our study examined the incidence of HO at our institution with one surgeon who performed direct anterior approach total hip arthroplasties (DAA THA). DAA THA is a popular approach with a reported learning curve of up to 100 cases.^16,17^ There is also a potential for significant variation surgeon to surgeon when it comes to soft tissue handling, use of special tables/retractors, and post-operative management.^11^ The authors aimed to quantify the overall incidence of H.O. amongst our cohort and compare this to the rates in the literature. Risk factors for development of H.O. were also examined and analyzed in the study.

## METHODS

Before data collection, IRB exempt determination was obtained from the Detroit Medical Center internal review board committee. Patients who had recived a DAA THA from 3/8/2016 to 1/22/2019 were included in the analytic sample. Additional inclusion criteria included: patient age 18 or older, minimum of three months postoperative follow up, and use of either ASA 325 mg. or ASA 81 mg. post-operatively for thromboembolic prophylaxis. Exclusion criteria included: patients who had received revision surgery, had prior hip surgery, infection after index procedure, or undergoing THA with any approach besides direct anterior.

The surgeon, (senior author DK) used a special table for each of his anterior hip patients, with a large C-arm for positioning of femoral and acetabular components. This surgeon was well beyond the stated learning curve (>100+ cases) for DAA THA. Dissection and all systematic releases for primary hips were consistent throughout the study period. A capsulotomy (opening of the anterior hip capsular tissue) was performed in each sample patient. An attempt to close the capsule tightly was performed at the conclusion of every case. Additionally, to evaluate contributing factors in the development of HO after DAA-THA, additional data points were collected on all patients who met inclusion criteria. Retrospective sociodemographic data such as age, gender, and race were also collected as well as clinical data regarding comorbidities such as diabetes, cardiovascular disease, osteoporosis, smoking status.

A manual medical record review was conducted by three upper level residents (PK, RD, SY) to investigate if administration of a NSAID was administered during the hospital stay; specifically, both drug type and dose were recorded. DVT prophylaxis, specifically the dose of ASA, was also recorded. Finally, HO was conducted by one senior resident (first author PK), using the Brooker Classification System.^18^ (Figures 1a-1c) Observing this system, Class 1 is described as islands of bone within the soft tissues about the hip. Class 2 includes bone spurs originating from the pelvis or proximal end of the femur, leaving at least 1 cm. between opposing bone surfaces. Class 3 consists of bone spurs originating from the pelvis or proximal end of the femur, reducing the space between opposing bone surfaces to less than 1 cm. Class 4 shows apparent bone ankylosis of the hip.^19^ Finally, if post-operative complications such as dislocation occurred this was included in our review of the EMR.

**Figure 1(a):**
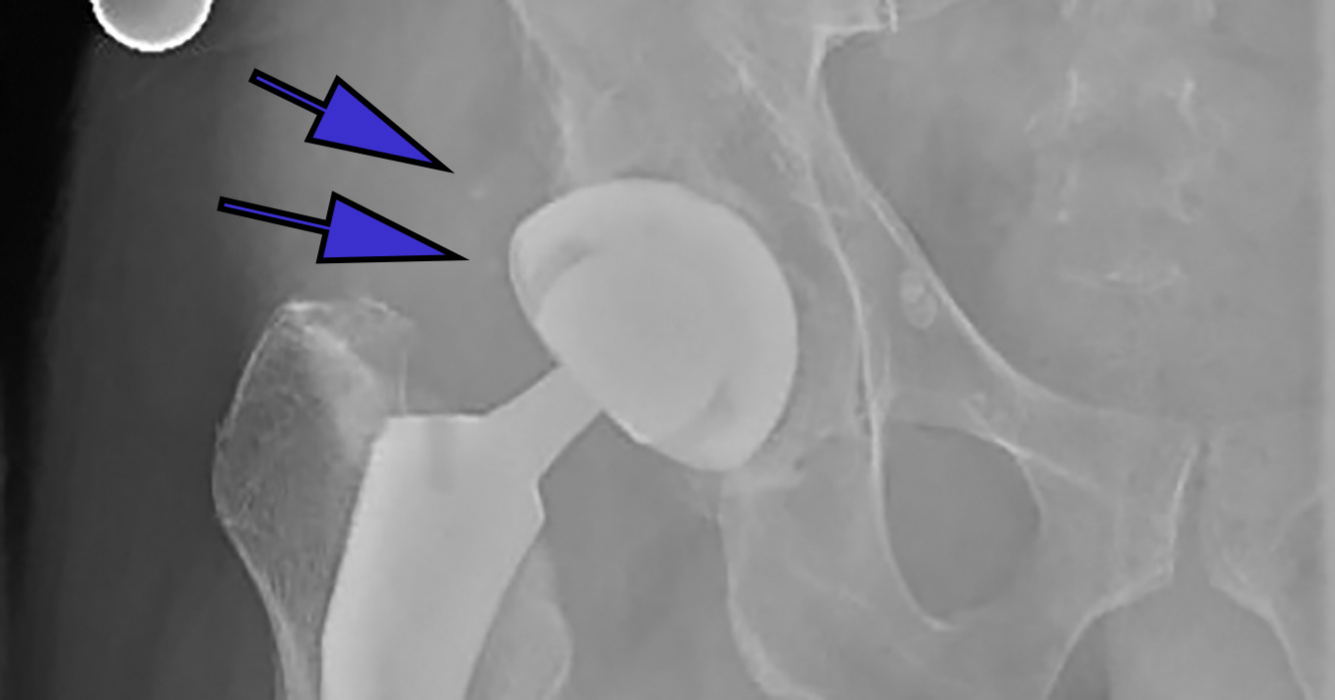
Anteroposterior (AP) radiographs of post-op total hip arthroplasties with differing classes of HO according to the Brooker classification Class 1: showing small islands of bone within the soft tissue

**Figure 1(b):**
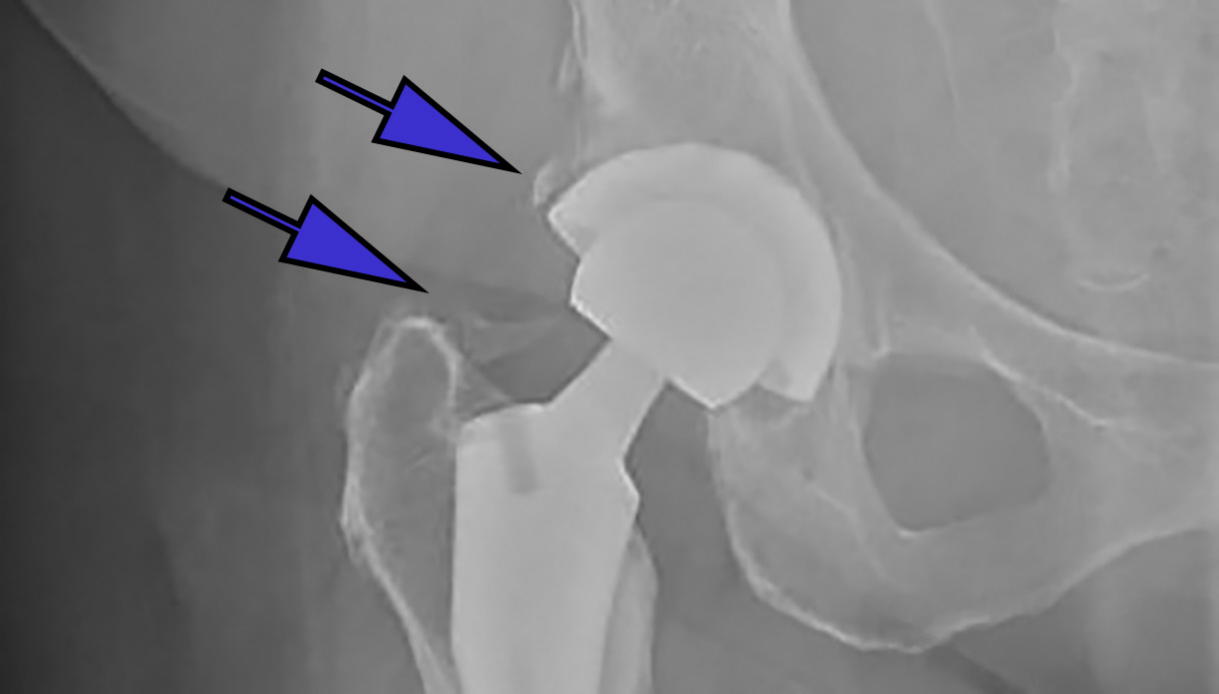
Anteroposterior (AP) radiographs of post-op total hip arthroplasties with differing classes of HO according to the Brooker classification Class 2: bone growth from the femur and pelvis leaving >1cm from end to end

**Figure 1(c):**
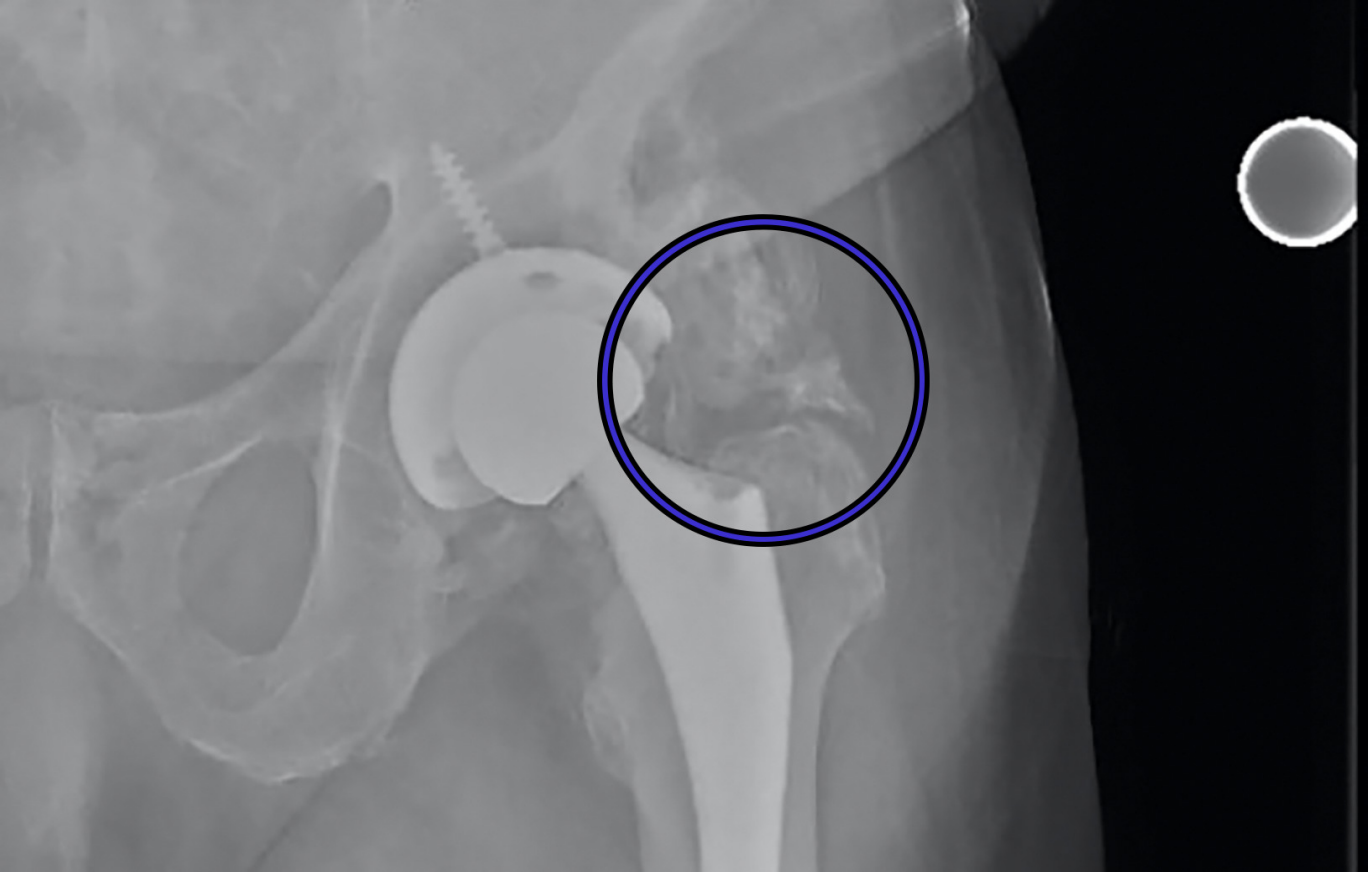
Anteroposterior (AP) radiographs of post-op total hip arthroplasties with differing classes of HO according to the Brooker classification Class 3: bone growth extending from femur and pelvis with <1cm between ends

Our experienced statistician (fifth author JB) used *SAS for Windows®, version 9.4* software for all study analyses. Categorical variables were examined with Chi-square tests where appropriate. Otherwise Fisher’s Exact tests were used. Age, BMI and follow up are provided as means +/- the standard deviation, followed by the minimum to maximum (range). Wilcoxon rank sum tests were used to analyze for possible relationship between these variables.

## RESULTS

A total of 45 patients met inclusion criteria and were included for analysis. All surgeries were performed by one surgeon at the single hospital setting with dates ranging from 3/8/2016 to 1/22/2019. There were 23 (51%) female and 22 (49%) male patients with an average age of 58 (+/- 9) years. Mean BMI of the cohort was 29 (+/- 4.65). A total of 38 patients identified as Caucasian (85%), 12 patients identified as African American (13%) and five patients were of other racial background (2%). The median follow-up time for final radiographic analysis was 35 weeks from the date of index surgery with no significant difference in length of final follow up found between the patients who developed HO and those who did not (P = 0.64).

The incidence of HO in our study was 12/45 (26.67%). Class I HO was found in eight patients (66.67%), Class II in one patient (8.33%), Class III in three patients (25%), and zero patients with Class IV. Postoperative NSAID use as needed was noted in 29 (64.4%) patients and there was no statistically significant relationship noted (P = 1.00) between the development of HO (8/12; 66.7%) and not developing HO (21/33; 63.6%). The mean BMI for those who developed HO was 29.3 (+/- 4.7) while those without had an average BMI of 29.7 (+/- 4.7) with no statistically significant difference noted between groups (P = 0.98).

Rates of pre-operative smoking status did reach statistical significance between the two sample subgroups with three (9.1%) of those patients without HO reporting history of smoking while six (50%) of those who did develop HO reporting a positive smoking history (P = 0.006). There were no significant differences found between the two subgroups with regards to age, sex, race, or diabetes (Table 1).

## DISCUSSION

The numerous surgical approaches for total hip arthroplasty have been a dominant topic in the literature with the more recent popularity of the direct anterior approach to the hip.^20^ Selected outcome indicators have included post-operative recovery time, narcotic use, and component malalignment as the focus of comparison studies between all approaches.^21,22^ There have been few studies on HO and the associated risk factors and prevention strategies. In this study, our goal was to combine variables studied in association with heterotopic ossification in the literature, which included surgical approach, use of ASA for thromboembolic prophylaxis, and surgical technique.

We found no significant difference between the two subgroups with regards to age, sex, race, or diabetes. We acknowledge that our sample may have failed to afford us an adequate level of statistical power to detect meaningful subgroup differences that may have been detected in a larger sample. We were, however, able to find a statistically significant difference in smoking rates between our two groups.

One might expect a decreased chance of HO in the smoking population due to the well-established negative/inhibitory effect of smoking on bone growth/healing.^23,24^ Studies of osteoblastic cells have shown both inhibition of protein synthesis as well as collagen formation with exposure to tobacco smoke.^25^ Clinical studies have shown conflicting results depending on the patient situation/clinical picture. In the setting of periprosthetic joint infection authors found an increased rate of HO in smokers, males, and patients with an increasing number of surgeries.^26^ In another, with a sample of military patients who underwent a traumatic amputation, there was no relationship found between smokers and the development of HO in matched samples.^27^ Literature on this relationship is certainly lacking.

In a 2014 study by Tippets et al, the authors investigated the incidence of HO in the direct anterior approach and found an overall incidence of 41.5%.^28^ In this study, there were two separate surgeons at independent hospitals included. Interestingly enough, they found a significant difference in the formation of HO between patients receiving ASA and Lovenox (25.7 v 47.5%, respectively).^28^ In a 2010 study by Cohn et al, a posterolateral approach was used exclusively, but similar results were found as patients receiving aspirin ASA had an incidence of 22.2%, compared to the Coumadin group at 51.7%, which was statistically significant.^8^

Coumadin, a vitamin K antagonist, can also be used for the prevention of venous thrombosis after orthopaedic surgery.^29^ Another mentioned pharmacologic therapy, Lovenox, is a low molecular weight heparin which can also be used in the prevention of DVT and PE after general and/or orthopaedic surgery.^30^

The reason for potential improvement in outcomes with use of concomitant NSAIDs has previously been studied.^31^ Although a consensus has not been reached on the exact mechanism, there are numerous theories. In THA, there is the process of reaming and broaching of the femoral canal and acetabulum, which releases bone and its precursor cells into the surgical window. Prostaglandin-E2 is thought to be involved in the process of HO, which explains the inhibition of this process with anti-inflammatory medications.^11^

Studies have shown that doses of indomethacin have been effective at preventing HO, with the optimal dosage being a topic of controversy.^32–34^ Radiation therapy is another common method of prophylaxis. In a randomized trial by Liu et al, the authors showed a decrease in HO formation in patients receiving 700cGy compared to doses of 400cGy without any increase in wound complications.^35^

In addition to the introduction of bone progenitor cells and growth factors (early stage cells and induction factors that lead to new bone formation) into the soft tissues from reaming, there is the possibility that the handling and dissection of soft tissues is the main culprit in HO formation. This theory was supported in 2001 by Sneath et al who found that immediate pulse lavage (repetitive pressurized irrigation) with 3L of normal saline did not decrease the rate of HO when compared to 500 mL of irrigation with a syringe.^36^

The idea that surgical handling of the tissues results in HO is especially interesting when approaches to the hip are discussed. In a retrospective case control study by Hurlimann et al, the incidence of HO was compared between approaches with 134 consecutive patients undergoing THA. Rates of HO were 31.3% (standard anterolateral), 20.9% (standard post lateral), 29.1% (minimally invasive anterior approach), and 18.7% (minimally invasive anterolateral).^37^ Unfortunately, there was no mention of post-op NSAID use or what thromboembolic prophylaxis was used during their study. Another study by Alijanipour et al, showed rates of HO at 36.1% for direct lateral and 19.4% for direct anterior indicating low rates of HO in the anterior approach.^38^

The variability between surgeons and the extent of their soft tissue releases and exposures can significantly impact the amount of damage to surrounding tissues.^38^ This is the reason we aimed to use one surgeon in our analyses. Our rate of HO in the DAA (26.7%) was similar to rates described in the literature ^28,37,38^ and we were able to keep these surgical variables constant.

**Table 1:** Demographic data and variables of interest with and without HO

	**No HO** **N=33**	**Presence of HO** **N=12**	**p-value**
**Age**			
Mean+/- Standard Deviation (median)	57 +/- 11 (57)	44 to 76	0.97
Minimum to Maximum	30 to 75	58 +/-9 (59)	
**BMI**			
Mean +/- SD (median)	29.7 +/- 4.7 (29)	29.3 +/- 4.7 (30.5)	0.98
Minimum to Maximum	21.6 to 39.6	20.6 to 35.2	
**Males**	14 (42.4%)	8 (66.7%)	0.19
**Race**			
African American	3 (9.1%)	3 (25%)	0.50
Caucasian	29 (87.9%)	9 (75%)	
Other	1 (3.0%)	0	
**Diabetes**	1 (3.0%)	0	1.00
**Preoperative Smoking Status (+)**	3 (9.1%)	6 (50%)	**0.006**
**DVT/PE PPx**			
ASA 325 mg. BID	14 (42.4%)	3 (25%)	0.62
ASA 81 mg. BID	19 (57.6%)	9 (75%)	
**As Needed NSAID use post-op**	21 (63.6%)	8 (66.7%)	1.00
**Follow up (# of weeks)**			
**Median (25 ^th^, 75 ^th^)**	35 (13, 52)	35 (13, 61)	0.64
**Minimum to Maximum**	12 to 122	12 to 96	

One limitation of our study was that our institution’s post-op DVT prophylaxis changed in August 2016 when the protocol changed from ASA 325 mg. BID to ASA 81 mg. BID. Although this introduced some level of variability, we believe these differing dosages of the same medication would have a minimal effect on the outcomes of this study. It has since been shown that in regard to thromboembolic prophylaxis, there may be no difference in development of symptomatic DVT after THA between low dose and full dose ASA.^39^ Similarly, we hypothesized that the small change in dose would not affect the rates of HO, which was supported in our study (p = 0.62)

All patients who received other modes of prophylaxis were excluded from the study. Our median follow up time was 35 weeks (range:12 - 122). This was largely due to the fact that the primary surgeon has follow up clinic visits at two, six, 12 weeks and one year after THA. At the 12-week visit, radiographs are generally performed and evaluated. Patients are then asked to follow up at one year for further evaluation, but per protocol, radiographs were only obtained if there were any concerns about the prosthesis clinically. This occasionally led to availability of radiographs at the 12-week office visit, but none at the one-year visit, which could explain the average follow up of 8-9 months. It is important to consider that HO typically arises within six weeks post-operatively but can progress until about six months post-operatively.^18^

While there were no NSAIDs prescribed at time of discharge from the hospital, there was an order for the NSAID Ketorolac/Toradol 15mg every six hours as needed in 29 (64.4%) patients while in the hospital. The majority of our sample patients received one dose of this medication in the immediate post-op period and did not require additional doses. Patients were not prescribed any NSAIDs at the time of discharge and there were no prescriptions given for NSAIDs from our office in the post-operative period. There was no difference in incidence of HO found between the patients receiving NSAIDs as needed while inpatient and those who did not, indicating that this small dose of PRN (as needed) medication was not likely clinically significant.

A major strength of our study was the use of one fellowship-trained arthroplasty surgeon who exclusively uses the DAA for his total hip arthroplasties. This reduces the amount of variability when it comes to the approach and handling of the soft tissue. It is our understanding that there have not been any studies looking exclusively at direct anterior approach with a standardized post-operative protocol including thromboembolic ASA prophylaxis.

## CONCLUSIONS

This is the first apparent study to examine the incidence of HO formation after DAA THA using exclusively ASA as DVT/PE prophylaxis. The incidence of HO was 26.7% and does not appear to be influenced by demographic factors including age, sex, race, or diabetes. Based on these results, there may be a correlation between smoking and HO formation after THA, although further research into this relationship is needed. Additionally, post-op NSAID use as needed while inpatient did not appear to influence HO formation following THA in this patient sample. In conclusion, the incidence of HO after DAA THA with post-op aspirin DVT/PE prophylaxis in our study is similar to that published in other research, although there remains a need for larger-sample prospective studies examining this topic.

### Conflict of Interest

The authors declare no conflict of interest.
